# The Compulsory Elevation of Doctors

**Published:** 1889-03-23

**Authors:** 


### The Hospital

A Weekly Institutional Journal of

Science, Medicine, Nursing, and Philanthropy.

*For Hospitals, Asylums, Medical Practitioners, Students, Nurses, Families, Parishes Congregations, and the Charitable Public.*

Vol. V. No. 130.] [March 23, 1889.

### The Compulsory Elevation of Doctors.

When three people attempt to stand upon a pedestal on which there is only room for two, one of them will either be pushed off and fall to the ground, or he will struggle upwards and stand on the shoulders of the other two. Fifty years ago the medical practitioner, who was also an apothecary, had a pedestal all to himself. It was neither very tall, nor very beautiful, nor very spacious at the summit; but such as it was he alone occupied it, with both right and possession on his side. In these days the pedestal has improved both in height and beauty of aspect, but it is not much more spacious at the top; and what is worst of all, two other persons often claim to share it with the unfortunate doctor. Those two persons are the chemist and the trained nurse. We need not here waste energy by discussing the justice or otherwise of their claims; but it may be profitable both for the public, for medical men, and for all concerned to devote a little space to the consideration of the spirit and movement of the age as it affects the broad question of the medical treatment of the sick.

Every person of substance when seriously ill has at least three orders of beings who keep watch and ward against the approach and assault of death. He has his medical attendant, his trained nurse, and his dispensing chemist. In quite recent times the doctor himself performed the functions of all these. For he always prepared and sent the medicine; and if he did not carry out every detail of nursing in person, he undertook all the more important parts of it, and gave the minutest instructions from day to day and from visit to visit concerning the minor circumstances. So much was this the case that many a doctor, who also happened to be a good nurse, was laughed at by the more cynical of his critics for being "an old woman." Yet that very peculiarity of character often endeared him to his patients and ensured his success. It is coming about now that persons of no substance are demanding the same high order of medical skill as their richer neighbours, and the same care and efficiency in nursing. Not only so, but such is the state of public opinion that the claim is very readily allowed, and all kinds of efforts are made by all classes of people to provide for the very poorest the best medical science and practical ability of the times, and also the best nursing. There is a sympathy with the sufferings of the meanest, and a value put upon their lives which has never been known at any previous time.

Now the spirit of the age, though a very impalpable entity, is a very resistless power. Medical men and others may be reminded of the period, by no means remote, when hand-made goods were replaced by the products of machinery, and when the introduction of machines of various kinds gave rise to bloody riots in many localities, and to a widespread resistance which almost assumed the proportions of a rebellion. Yet machinery won the day; and in spite of their poverty, their starvation, and their despair, the workers of the manufacturing districts were compelled, by the steady march of events, to accept the inevitable, and to adapt themselves as best they could to the changed conditions. It is quite certain that what may be called the business aspect of medical practice is undergoing a marked and rapid change. At present the doctor's affairs of this class are in a state of solution; but the final form into which they will crystallise it is not very difficult to foresee. Every practitioner will do well to open his eyes and gradually to adapt himself with intelligent willingness to changes which no resistance, however blind or obstinate, can avert.

Circumstances infallibly point to this, that the doctor must be prepared to give up a portion of his ground to others. The chemist and the trained nurse have established the fact of their value, as well as of their existence. The development of new organs gives rise to a higher degree of specialisation of formation. If the physician is driven from a lower ground it is only that he may occupy a higher; if two claimants insist upon sharing his pedestal, it is only that he may stand on the shoulders of both, and perform by their aid feats of vision and of skill which without such aid would be well-nigh impossible. The demand upon the practitioner of the future will be for higher diagnostic powers, a clearer faculty of prevision and prognosis, more judgment and skill in prescribing, and greater authority and decision in guiding and directing others. The days of the scienceless apothecary are numbered, the morning of the family physician has already dawned. By a process of "natural selection" the profession is everywhere dividing itself by sharp lines of cleavage into two classes. There is the class of cheap dispensers, who take shops, advertise like any other tradesman, and who to all intents and purposes belong to the lower ranks of chemists and druggists. On the other hand is the more numerous class of educated men, who, while not suffering themselves to be bound by the chains of a merely formal and meaningless etiquette, stand firmly by the best traditions of the past and band themselves together into a great army for the advancement of their science, the improvement of their art, and the maintenance of a noble enthusiasm and a generous *ésprit de corps.*

The process of adapting themselves to their new conditions cannot be carried out by the doctors without a good deal of privation and suffering. For just as the introduction of machinery made the demand for human labour smaller, so is the rise of the chemist and of the trained nurse diminishing the necessity for the frequent calls and the close personal supervision of the medical attendant. In acute and dangerous cases the morning, and in some instances the evening and mid-day call will be needed as before. But in lingering and chronic illnesses, where the trained nurse is always on the spot to watch and to report, and to send a swift messenger in the event of need, the daily visit will give place to the bi-weekly, or even to the less frequent call. Of course when visits are fewer in number fees will necessarily rise; but they can never rise so high as to compensate for the more frequent calls of former days. The doctor's income, already far too small, must become still smaller, and his struggle for survival still more severe. In due time, however, the reward will come, and the public as well as the medical profession will share in its advantages. As frequent calls upon patients become less indispensable, and fees rise, the doctor will be able to live without such a tremendous strain and expenditure of energy as he is now compelled to endure. He will have both leisure and strength to do that without which the ablest man in the world cannot keep pace with the best progress of the times. He will be able to read, and to study, and, what is as necessary as either, to meet his professional brethren in frequent and friendly conference and discussion.

The public at large have a much more immediate and practical interest in this question than they suppose. The medical profession has always offered a career to a certain proportion of the clever youth of the country. With the rise of the trained nurse and the educated chemist the field for doctors is necessarily very much limited in area, and the prospects of success, or even of a livelihood, are correspondingly diminished. There seems to be as yet no general comprehension of this fact; because young men are flocking to the gates of the profession in increasing numbers year by year. Of course, a medical education can by no possibility do those young men any harm—on the contrary, it may be of very great service to them. But it is certain that many of them will never make use of it as a means of earning a livelihood, because it will not be worth their while to do so. To all those, therefore, whose means are limited, and whose resources will not enable them to qualify for a second profession if the first they choose should fail them, the advice is given with much seriousness—to think well before they adventure themselves upon a medical career. The number of candidates for medical openings is rapidly increasing. The number of openings is, owing to the causes we have indicated, still more rapidly diminishing. When men increase in excess of the demand for their services there is but one result possible—starvation for many, and keen competition for all.

